# Impact of Neuraminidase Inhibitor Treatment on Outcomes of Public Health Importance During the 2009–2010 Influenza A(H1N1) Pandemic: A Systematic Review and Meta-Analysis in Hospitalized Patients

**DOI:** 10.1093/infdis/jis726

**Published:** 2012-11-29

**Authors:** Stella G. Muthuri, Puja R. Myles, Sudhir Venkatesan, Jo Leonardi-Bee, Jonathan S. Nguyen-Van-Tam

**Affiliations:** 1 Health Protection and Influenza Research Group, Division of Epidemiology and Public Health; 2 Division of Epidemiology and Public Health, University of Nottingham, United Kingdom

**Keywords:** neuraminidase inhibitors, mortality, critical care admission, pneumonia, systematic review, meta-analysis

## Abstract

***Background.*** The impact of neuraminidase inhibitor (NAI) treatment on clinical outcomes of public health importance during the 2009–2010 pandemic has not been firmly established.

***Methods.*** We conducted a systematic review and meta-analysis, searching 11 databases (2009 through April 2012) for relevant studies. We used standard methods conforming to Preferred Reporting Items for Systematic Reviews and Meta-Analyses (PRISMA) guidelines. Pooled odds ratios (ORs) and 95% confidence intervals (CIs) were estimated using random effects models.

***Results.*** Regarding mortality we observed a nonsignificant reduction associated with NAI treatment (at any time) versus none (OR, 0.72 [95% CI, .51–1.01]). However we observed significant reductions for early treatment (≤48 hours after symptom onset) versus late (OR, 0.38 [95% CI, .27–.53]) and for early treatment versus none (OR, 0.35 [95% CI, .18–.71]). NAI treatment (at any time) versus none was associated with an elevated risk of severe outcome (OR, 1.76 [95% CI, 1.22–2.54]), but early versus late treatment reduced the likelihood (OR, 0.41 [95% CI, .30–.56]).

***Conclusions.*** During the 2009–2010 influenza A(H1N1) pandemic, early initiation of NAI treatment reduced the likelihood of severe outcomes compared with late or no treatment.

***PROSPERO Registration.*** CRD42011001273.

**(See the editorial commentary by Aoki and Hayden, on pages 547–9.)**

The neuraminidase inhibitors (NAIs), oseltamivir and zanamivir are licensed for the treatment of influenza A and B. Before the 2009–2010 pandemic, evidence from randomized trials suggested modest reductions in time to alleviation of symptoms and symptom severity [1–4] and possibly a reduction in antibiotic use for secondary complications [5–7]. Further evidence from methodologically weaker observational studies, derived mainly from prepandemic data (seasonal influenza), suggests that oral oseltamivir reduces mortality by about 75%, hospitalization by 25% and symptom duration compared with no treatment, with broadly similar findings for zanamivir, based on fewer studies [8].

Despite limited usage since launch, except in Japan, both drugs, especially oseltamivir, were widely stockpiled for pandemic purposes and subsequently deployed during the influenza A(H1N1)pdm09 pandemic. A subsequent analysis of oseltamivir safety data published by F. Hoffman–La Roche estimated that 18.3 million individuals worldwide received the drug during the pandemic period between 1 May and 31 December 2009 [9], and data from the United States shows that 97.5% of prescriptions for NAIs during the pandemic period were for oseltamivir [10].

Published studies from the recent pandemic period suggest that early (≤48 hours after symptom onset) versus “late” (delayed >48 hours after symptom onset) treatment of healthy and at-risk adults reduced the likelihood of hospitalization or requirement for critical care [11–15]. Similarly, a small number of studies suggest that increased in-hospital mortality might be related to the late initiation of NAI therapy [16–19]. However, many studies are too small to produce conclusive individual findings; some adjust for possible confounders, but most do not.

Considerable uncertainty remains among public health policy-makers and governments regarding the public health benefits of NAI usage during the 2009–2010 pandemic. We therefore present a systematic review and meta-analysis of studies specifically from that period, assessing the impact of NAI treatment in hospitalized patients on mortality, requirement for critical care, and influenza-related pneumonia.

## METHODS

### Eligibility Criteria and Assessment

#### Types of Studies

We included all comparative epidemiological studies (case series, case-control, and cohort studies) and randomized controlled trials conducted during the time period between 1 March 2009 (Mexico), or 1 April 2009 (rest of the world) until the WHO declaration of the end of the pandemic (10 August, 2010); assessing the association between NAI treatment and clinical outcomes. Studies with <10 participants were excluded.

#### Types of Participants

Subjects of all ages hospitalized with a clinical or laboratory diagnosis of A(H1N1)pdm09.

#### Types of Interventions

Treatment with an NAI (oseltamivir, zanamivir and peramivir [20]) administered via any route for A(H1N1)pdm09. Articles reporting combined results with other influenza virus types, subtypes, and strains were excluded.

#### Types of Outcome Measures

Mortality, admission to critical care, and influenza-related pneumonia.

### Search Strategy

We searched Medline, EmBase, CINAHL, CAB Abstracts, ISI Web of Science, PubMed UK, PubMed central, Scopus, WHO regional indexes, LILAC, and J-STAGE (to 19 April 2012), imposing no language restrictions. Further studies were also identified from scanning reference lists of identified studies and through contact with subject area experts (via J. S. N. V. T.). We used Boolean logic and core search terms relating to pandemic influenza (including *influenza A virus* OR *H1N1 subtype* OR *swine origin influenza AH1N1* virus) AND exposure of interest that is, antiviral drugs (including *neuraminidase inhibitors* OR *oseltamivir* OR *zanamivir* OR *peramivir*) AND clinical outcome measures (including *pneumonia, or critical care/intensive care, or mortality*). Our detailed search strategy is shown in Supplementary Table 1.

### Screening, Data Extraction, and Quality Assessment

Titles, abstracts, and full texts of identified studies were screened independently by 2 reviewers (S. G. M., S. V.) with differences being resolved through discussion with a third reviewer (P. R. M.). Data from included studies were independently extracted by 2 investigators (S. G. M. and S. V.) using a previously piloted data extraction form, and scored for methodological quality using the Newcastle-Ottawa Quality Assessment Scale (NOS) [21]. This scale awards a maximum score of 9 points to each included study based on representativeness of the cohort, adjustment for confounders and assessment of the outcome/exposure. Where relevant and possible, supplementary data were sought from corresponding authors of included studies. Differences in quality assessment were resolved by referral to a third investigator (P. R. M.).

### Data Analysis

Results from individual studies were extracted directly as odds ratios (ORs), with 95% confidence intervals (CIs; or standard errors), or as tabulated data, from which ORs were estimated based on adjustment for the greatest number of covariates possible in each analysis. The data were pooled using random effects meta-analysis. Separate analyses were performed for the following 3 treatment exposures: NAI treatment (irrespective of timing) versus none; early NAI treatment (≤48 hours after symptom onset) versus late treatment (delayed >48 hours after symptom onset); and early NAI treatment versus no treatment. Heterogeneity between studies was assessed using the *I*^2^ statistic [22]; when at least moderate (*I*^2^ > 50%), subgroup analyses were conducted to explore the effects of age; ascertainment of A(H1N1)pdm09 diagnosis; special health states (eg, pregnancy, intensive care unit admission, pneumonia); and study quality (Newcastle-Ottawa Quality Assessment Scale >6 vs ≤6). Publication bias was determined using funnel plots and Egger's tests [23]; all analyses were conducted using Stata v11.2 software (StataCorp).

### Protocol and Registration

We adhered to the recommendations for Preferred Reporting Items for Systematic Reviews and Meta-Analyses (PRISMA) [24], and the protocol is published in the National Institute for Health Research international prospective register of systematic reviews (PROSPERO) [25].

## RESULTS

### Study Selection and Characteristics

**Figure 1.**
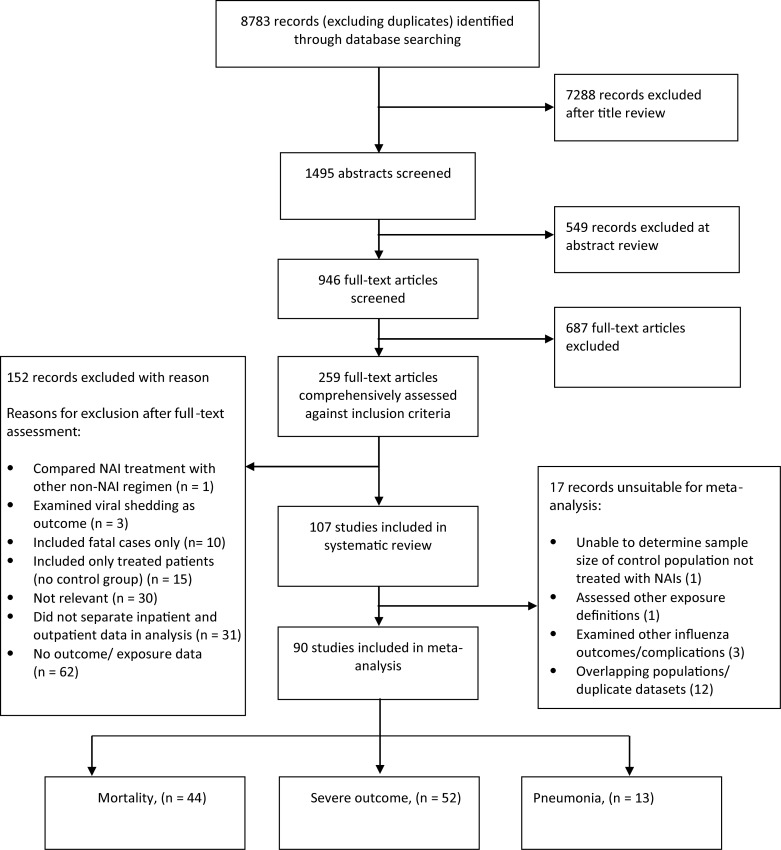
Summary of article selection process. Abbreviation: NAI, neuraminidase inhibitor.

Of 8783 records identified from electronic searches, 1495 titles were judged potentially relevant, and their abstracts screened for relevance, yielding 259 full-text records. After these were assessed, 107 articles were eligible (Figure 1). A full reference list of eligible articles is provided in Supplementary Table 2. Of the 107 articles, 53 assessed mortality, 59 assessed severe outcome (defined as critical care admission or death) and 14 assessed A(H1N1)pdm09-related pneumonia (Supplementary Table 2). Seventeen articles could not be included in the meta-analyses because they were partially or completely included as part of a national surveillance dataset or larger study earmarked for inclusion within the overall meta-analysis (Figure 1); reasons for exclusions are provided in Supplementary Table 3.

Characteristics of the 90 studies eligible for meta-analyses are summarized in Table 1. Eighty (89%) reported exclusively laboratory-confirmed diagnoses, positive by A(H1N1)pdm09-specific polymerase chain reaction (PCR) or positive by PCR for influenza A but nontypeable for human subtypes H1 (seasonal); 8 (9%) studied hospitalized patients with confirmed, probable, or suspected A(H1N1)pdm09 infection. Two studies reported A(H1N1)pdm09 cases but did not specify methods of diagnosis.

**Table 1.** Summary of 90 Studies Included in Meta-Analysis, by Outcome Measure

Outcome Measure^a^	Mortality	Severe Outcome^b^	Pneumonia
Studies, No.	44	52	13
Total sample size, No. of patients	23 723	31 428	3271
Male patients, No.^c^	11 558	13 608	1602
Age range, y	<1 to 93	<1 to 91	<1 to 93
Population groups, No. of studies			
Mixed age groups	20	21	5
Adults	8	8	3
Children	5	10	2
Pregnant women	4	4	3
Other	7	9	…
Regions, No. of studies			
North America	9	18	2
Latin America	9	4	2
Europe and Australia/New Zealand	10	18	2
Asia-Pacific	14	12	7
Others	2	…	…
A(H1N1)pdm09 diagnosis, No. of studies			
Laboratory confirmed	37	49	11
Laboratory confirmed or clinically diagnosed cases	6	3	1
Confirmed cases but method of confirmation not stated	1	…	1
A(H1N1)pdm09 diagnosis, No. of patients			
Laboratory confirmed	15 998	29 574	3059
Laboratory confirmed or clinically diagnosed cases	7707	1854	31
Confirmed cases but method of confirmation not stated	18	…	181
Antiviral agents used, No. of studies^d^			
Oseltamivir only	23	24	9
NAI only	8	10	4
NAI and non-NAI antiviral^e^	5	5	…
NAI drug name not specified	8	13	…
Exposure comparison, No. of studies^f^			
Any NAI vs none	23	29	6
Early NAI vs late NAI	27	30	11
Early NAI vs none	9	13	4
Early NAI vs late NAI or no treatment	…	2	…
Preadmission NAI vs no preadmission NAI	2	3	…
Patients, No.^g^			
Treated with any NAI	14 920	25 246	2964
Treated with early NAI	3652	5583	1085
Treated with late NAI	6549	11 993	1226
Untreated with NAI	1738	4818	186
Studies adjusting for potential confounders, No. (%)	8 (18)	11 (21)	0 (0)
NOS score, median (range)	6 (4–9)	6 (4–9)	5 (4–8)

Abbreviations: NAI, neuraminidase inhibitor; NOS, Newcastle-Ottawa Quality Assessment Scale.

^a^ Some studies examined multiple outcomes.

^b^ Severe outcome was defined as critical care admission or death.

^c^ The breakdown by sex was unknown in a small number of studies (3 each in the mortality and severe outcome analyses).

^d^ Overall, 7 studies provided information on combined oseltamivir and peramivir use in 14 patients (see Supplementary Table 2).

^e^ Overall, 8 studies reported combined use of NAI and non-NAI (rimantadine, amantadine, or ribavarin) therapy (n = 77 patients; see Supplementary Table 2).

^f^ Some studies examined multiple exposure comparisons. Early NAI was defined as treatment beginning within ≤2 d after symptom onset; late NAI, treatment beginning >2 d after symptom onset.

^g^ Best estimates of numbers of patients (some publications provided insufficient data on patient numbers by treatment category).

Forty-five studies (50%) in the meta-analyses reported treatment with oseltamivir only, 21 (23%) reported treatment with NAIs (oseltamivir, zanamivir, and/or peramivir), 8 (9%) reported monotherapy with NAI and non-NAI antiviral drugs (amantadine, rimantadine, ribavirin), and in 16 studies (18%) the name of NAI drug was not specified. Overall, 34 895 patients were treated with an NAI, of whom 14 (0.0004%), across 7 studies, were treated with peramivir either alone or as dual therapy with oseltamivir. Seventy-seven patients (0.002%) across 8 studies also received combined therapy using NAI and non-NAI antiviral drugs (typically NAI plus either adamantane or ribavirin). Because we did not have access to individual-level raw data, it was not possible to exclude such patients without sacrificing eligible whole studies.

### Meta-Analysis Findings

#### Mortality

**Figure 2.**
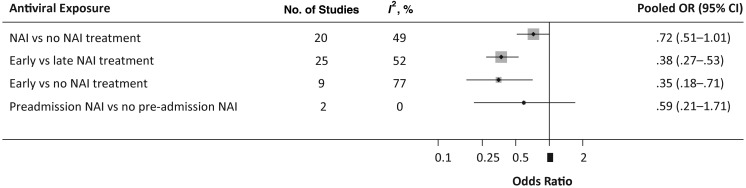
Summary of pooled analyses from studies examining mortality. Abbreviations: CI, confidence interval; NAI, neuraminidase inhibitor; OR, odds ratio.

Fifty-three studies presented data on the association between NAI treatment and mortality. Nine studies were unsuitable for meta-analyses and were excluded (Supplementary Tables 2 and 3). Analyses of the remaining 44 are summarized in Figure 2. The pooled analysis of 20 studies comparing NAI treatment (at any time) versus none revealed a nonsignificant reduction in risk of mortality (OR, 0.72 [95% CI, .51–1.01]), with moderate statistical heterogeneity (*I*^2^, 49%) and no evidence of publication bias (Egger's test, *P* = .894). Moreover, meta-analysis of 2 studies examining preadmission NAI treatment versus no preadmission NAI in subsequently hospitalized patients did not find a statistically significant reduction in mortality (OR, 0.59 [95% CI, .21–1.71]) (Table 2).

**Table 2.** Summary of Results (Random Effects Model) Including Subgroup Analyses for Mortality, Severe Outcome, and A(H1N1)pdm09-Related Pneumonia

Hospitalized Patients	Studies Included in Analysis, No.	Pooled OR (95% CI)	*I*^2^, %	References^a^
Mortality (Died vs Survived)				
NAI vs no NAI treatment (overall)	20	.72 (.51–1.01)	49	[3–5, 16, 17, 26, 38, 41, 43, 62, 63, 69, 78, 83, 85, 92, 94, 97, 99, 104]
Unadjusted studies	18	.73 (.53–1.00)	44	[4, 5, 16, 17, 26, 38, 41, 43, 62, 63, 69, 78, 83, 85, 92, 97, 99, 104]
Adjusted studies	2	1.22 (.01–172.42)	85	[3, 94]
A(H1N1)pdm09 diagnosis				
Laboratory confirmed cases	16	.77 (.54–1.08)	48	[5, 17, 26, 38, 41, 43, 62, 63, 69, 78, 83, 85, 92, 97, 99, 104]
Laboratory confirmed or clinically diagnosed	4	.50 (.14– 1.78)	54	[3, 4, 16, 94]
Mixed age groups	12	.75 (.50–1.13)	61	[3–5, 26, 38, 62, 69, 85, 88, 92, 94, 104]
Adults	7	.43 (.20–.97)	63	[16, 37, 43, 63, 85, 96, 104]
Children	6	.72 (.36–1.44)	12	[17, 41, 85, 97, 99, 104]
Pregnant women	1	.34 (.14–.81)	…	[78]
Patients with pneumonia	5	.74 (.13–4.28)	70	[43, 83, 88, 97, 104]
ICU patients	8	.61 (.41–.90)	5	[3, 16, 17, 41, 63, 78, 92, 99]
Others	…	…	…	[63]
Preadmission NAI treatment vs no preadmission NAI treatment	2	.59 (.21–1.71)	0	[26, 93]
Early treatment vs late treatment (overall)	25	.38 (.27–.53)	52	[2, 8, 13, 21, 26, 31, 32, 36, 37, 42, 49, 50, 52, 58, 65, 69, 72, 78, 84, 90, 92, 101, 102, 104, 107]
Unadjusted studies	23	.35 (.24–.51)	53	[2, 8, 13, 21, 26, 31, 32, 36, 37, 42, 49, 50, 52, 58, 69, 78, 84, 90, 92, 101, 102, 104, 107]
Adjusted studies	2	.61 (.31–1.19)	26	[65, 72]
A(H1N1)pdm09 diagnosis				
Laboratory confirmed cases	23	.37 (.26–.52)	53	[2, 8, 13, 21, 26, 31, 32, 37, 42, 49, 50, 52, 58, 65, 69, 72, 78, 84, 90, 92, 102, 104, 107]
Laboratory confirmed or clinically diagnosed cases	2	.33 (.03–3.73)	61	[36, 101]
Mixed age groups	14	.51 (.36–.72)	50	[13, 26, 32, 36, 37, 65, 69, 72, 84, 85, 90, 92, 102, 104]
Adults	10	.41 (.28–.59)	0	[8, 21, 37, 42, 49, 58, 82, 85, 104, 107]
Children	4	.37 (.20–.68)	0	[50, 52, 85, 104]
Pregnant women	4	.09 (.04–.21)	0	[31, 78, 98, 101]
ICU patients	9	.33 (.17–.64)	59	[8, 21, 36, 42, 52, 78, 92, 98, 102]
Patients with pneumonia	1	.53 (.19–1.5)	…	[104]
Others	…	…	…	[20, 32]
Early treatment vs no treatment (overall)	9	.35 (.18–.71)	77	[26, 31, 37, 65, 69, 78, 85, 92, 104]
Mixed age groups	6	.43 (.23–.80)	69	[26, 65, 69, 85, 92, 104]
Adults	5	.22 (.07–.66)	73	[31, 37, 78, 85, 104]
Children	2	.12 (.02–.76)	0	[85, 104]
Pregnant women	2	.07 (.02–.20)	0	[31, 78]
ICU patients	2	.28 (.02–3.88)	94	[78, 92]
Severe Outcome (Required Critical Care or Died vs Hospitalized and Survived)				
NAI vs no NAI treatment (overall)	23	1.76 (1.22–2.54)	86	[2, 5, 6, 9, 10, 18, 22, 24, 26, 27, 30, 33, 35, 45, 55, 61, 62, 64, 70, 77, 86, 95, 100]
Unadjusted studies	23	1.76 (1.22–2.54)	86	[2, 5, 6, 9, 10, 18, 22, 24, 26, 27, 30, 33, 35, 45, 55, 61, 62, 64, 70, 77, 86, 95, 100]
Mixed age groups	13	1.68 (1.05–2.70)	89	[2, 5, 6, 10, 18, 19, 26, 30, 55, 62, 64, 70, 77]
Adults	5	1.26 (.64–2.46)	60	[19, 45, 61, 67, 104]
Children	12	2.97 (1.81–4.89)	38	[9, 19, 22, 24, 27, 33, 53, 71, 74, 86, 95, 104]
Pregnant women	2	2.41 (1.71–3.39)	0	[14, 70]
Other	…	…	…	[95, 100]
Preadmission NAI treatment (before hospital admission)	3	.51 (.29–.89)	0	[26, 28, 93]
Early treatment vs late treatment (overall)	24	.41 (.30–.56)	82	[5, 8, 12, 13, 15, 18, 22, 24, 26, 30–32, 40, 44, 49, 58, 61, 64, 65, 70, 72, 73, 101, 105]
Unadjusted studies	19	.45 (.31–.66)	82	[5, 8, 13, 15, 18, 22, 24, 26, 32, 40, 44, 49, 58, 61, 64, 70, 73, 101, 105]
Adjusted studies	5	.33 (.19–.55)	77	[12, 30, 64, 65, 72]
Mixed age groups	11	.44 (.31–.62)	86	[5, 12, 13, 18, 26, 30, 44, 64, 65, 70, 72]
Adults	8	.63 (.38–1.02)	69	[8, 40, 47, 49, 58, 61, 66, 104]
Children	6	1.01 (.58–1.96)	57	[22, 24, 59, 71, 73, 104]
Pregnant women	4	.16 (.04–.60)	90	[15, 31, 70, 101]
Patients with pneumonia	3	.51 (.11–2.36)	91	[12, 66, 104]
Other (ARDS, diabetics, cancer, HIV)	…	…	…	[32, 100, 105, 106]
Early treatment vs no treatment (overall)	11	.94 (.50–1.76)	93	[5, 18, 24, 26, 30, 31, 61, 64, 65, 70, 73]
Mixed age groups	5	1.58 (.70–3.58)	96	[18, 26, 30, 70, 104]
Adults	2	1.01 (.18–5.74)	83	[61, 104]
Children	3	5.77 (.83–40.29)	79	[24, 71, 104]
Pregnant women	2	.59 (.07–5.19)	95	[31, 70]
Patients with pneumonia	1	3.77 (1.78–7.96)	…	[104]
Diabetics	1	.18 (.05–.61)	…	[100]
Early treatment vs late treatment or no NAI	2	.27 (.04–2.00)	23	[38, 39]
Pneumonia vs No Pneumonia				
NAI treatment vs none (overall)	6	2.29 (1.16–4.53)	26	[1, 33, 57, 60, 81, 108]
Unadjusted studies	6	2.29 (1.16–4.53)	26	[1, 33, 57, 60, 81, 108]
A(H1N1)pdm09 diagnosis				
Laboratory confirmation	5	2.83 (1.65–4.85)	0	[1, 33, 57, 81, 108]
Not specified	1	.26 (.02–3.04)	…	[60]
Pneumonia confirmation				
Chest radiographs	4	2.72 (1.54–4.82)	3	[1, 33, 81, 108]
Not specified	2	1.17 (.07–20.09)	66	[57, 60]
Mixed age groups	3	2.01 (.57–7.11)	21	[1, 57, 108]
Adults	2	1.10 (.12–10.16)	66	[60, 81]
Children	1	3.53 (1.63–7.66)	…	[33]
Pregnant women	1	.26 (.26–3.04)	…	[60]
Early treatment vs late treatment (overall)	11	.35 (.24–.50)	50	[1, 11, 49, 51, 57, 60, 76, 79, 81, 101, 103]
Unadjusted studies	10	.37 (.23–.58)	55	[1, 11, 49, 51, 57, 60, 76, 79, 101, 103]
Adjusted studies	1	.29 (.19–.45)	…	[81]
A(H1N1)pdm09 diagnosis				
Laboratory confirmation	9	.37 (.25–.55)	57	[1, 11, 49, 51, 57, 76, 79, 81, 103]
Laboratory and/or clinical confirmation	1	.19 (.02–1.78)	…	[101]
Not specified	1	.12 (.02–.66)	…	[60]
Pneumonia confirmation				
Chest radiographs	8	.36 (.24–.53)	58	[1, 49, 51, 76, 79, 81, 101, 103]
Not specified	3	.24 (.06–1.05)	38	[11, 57, 60]
Mixed age groups	3	.35 (.11–1.08)	76	[1, 57, 76]
Adults	7	.35 (.25–.47)	14	[11, 49, 51, 60, 81, 101, 103]
Children	1	.81 (.25–2.63)	…	[79]
Pregnant women	3	.31 (.04–.45)	0	[11, 60, 101]
ICU patients	2	.05 (.01–.20)	0	[11, 76]
Early treatment vs no treatment (overall)	4	.73 (.27–2.02)	48	[1, 57, 60, 81]
Mixed age groups	2	1.00 (.11–9.05)	69	[1, 57]
Adults	2	.62 (.11–3.89)	54	[60, 81]
Children	0	…	…	…
Pregnant women	1	.18 (.02–2.02)	…	[60]
Early treatment vs late treatment or no NAI	1	6.67 (2.61–17.06)	…	[40]

Abbreviations: ARDS, acute respiratory distress syndrome; CI, confidence interval; HIV, human immunodeficiency virus; ICU, intensive care unit; NAI, neuraminidase inhibitor; OR, odds ratio.

^a^See reference list in Supplementary Table 2.

Separate meta-analyses showed that early NAI treatment versus late (25 studies) was associated with a significant reduction in mortality (OR, 0.37 [95% CI, .27–.53]; *I*^2^, 52%), although there was evidence of asymmetry in tests for publication bias (Egger's test, *P* = .004). Pooled analyses for early NAI therapy compared with no treatment (9 studies) also found a significant reduction in mortality (OR, 0.35 [95% CI, .18–.71]; *I*^2^, 77%; Egger's test, *P* = .142). The high level of heterogeneity in this meta-analysis was partly attributable to the heterogeneous populations. Our subgroup analysis based on subpopulations found no evidence of heterogeneity for studies in children or pregnant women but high heterogeneity in intensive care unit–based studies (Table 2).

#### Severe Outcome (Critical Care Admission or Death)

**Figure 3.**
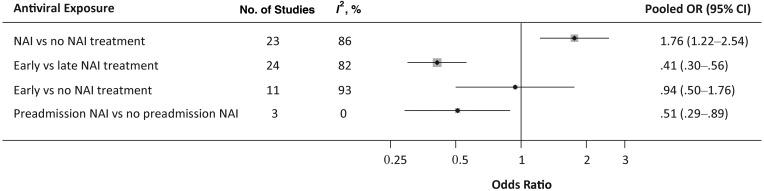
Summary of pooled analyses from studies examining severe outcome. Abbreviations: CI, confidence interval; NAI, neuraminidase inhibitor; OR, odds ratio.

Using a composite variable for “severe outcome” based on receiving critical care or death, 59 studies reported this outcome of which 52 were suitable for inclusion in meta-analyses; these are summarized in Figure 3 and Table 2. For NAI treatment (at any time) versus none (23 studies), a statistically significant increase in severe outcomes with NAI therapy was observed (OR, 1.76 [95% CI, 1.22–2.54]; *I*^2^, 86%; Egger's test, *P* = .036). We pooled 3 studies providing data on preadmission NAI use in hospitalized patients and found a statistically significant reduction in severe outcomes compared with no preadmission NAI (OR, 0.51 [95% CI, .29–.89]; *I*^2^, 0%; Egger's test, *P* = .46). Early NAI treatment compared with late (24 studies) also significantly reduced the likelihood of severe outcome (OR, 0.41 [95% CI, .30–.56]; *I*^2^, 82%; Egger's test, *P* = .016); however, early NAI treatment versus none (11 studies) revealed no statistically significant decrease in the likelihood of severe outcome (OR, 0.94 [95% CI, .50–1.76]; *I*^2^, 93%; Egger's test, *P* = .023). Two studies that assessed early NAI treatment versus late or none (combined) also revealed no significant reduction in severe outcomes (OR, 0.27 [95% CI, .04–2.00]; *I*^2^, 23%; Egger's test, not calculable; Table 2). Findings from all of these analyses were subject to high levels of heterogeneity (*I*^2^ > 75%) which were neither explained by subgroup analyses (Table 2) nor attributable to methodological quality (data not shown).

#### Pneumonia Associated With A(H1N1)pdm09 Infection

Fourteen studies reported data on hospitalized patients with A(H1N1)pdm09 infection and documented the presence or absence of pneumonia . Most reported radiographic pneumonia, whereas 3 did not provide information on ascertainment; the latter were still included in the meta-analysis but apportioned lower scores during quality assessment (Table 2).

**Figure 4.**
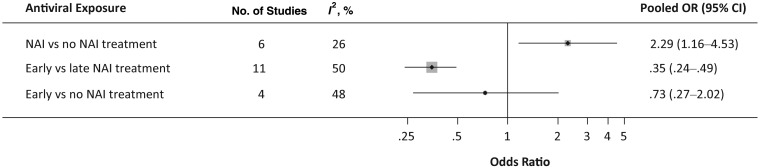
Summary of pooled analyses from studies examining A(H1N1)pdm09-associated pneumonia. Abbreviations: CI, confidence interval; NAI, neuraminidase inhibitor; OR, odds ratio.

The meta-analysis based on 13 articles is summarized in Figure 4. The pooled analysis comparing NAI treatment (at any time) versus none (6 studies) revealed a significantly increased likelihood of pneumonia associated with NAI treatment (OR, 2.29 [95% CI, 1.16–4.53]; *I*^2^, 26%; Egger's test, *P* = .282). However, early versus late treatment (11 studies) significantly reduced the likelihood of pneumonia (OR, 0.35 [95% CI, .24–.50]; *I*^2^, 50%; Egger's test, *P* = .646). A comparison between early treatment and none (4 studies) revealed no statistically significant decrease in the likelihood of pneumonia (OR, 0.73 [95% CI, .27–2.02]; *I*^2^, 48%; Egger's test, *P* = .826).

One pneumonia study (reference 40 in Supplementary Table 2) was unsuitable for inclusion in any of the pooled analyses because treatment exposure was measured as early versus late or none (combined). This study showed early oseltamivir treatment to be associated with a significantly increased likelihood of pneumonia (unadjusted OR, 6.67 [95% CI, 2.61–17.06]; *P* < .001).

## DISCUSSION

### Mortality

Overall, our meta-analyses suggest that NAI treatment of A(H1N1)pdm09 in hospitalized cases reduced mortality. Although comparison of treatment (at any time) with none revealed a 28% nonsignificant reduction in mortality, when comparing early with late treatment we observed a significant 63% reduction in mortality, albeit with significant publication bias. Finally, we noted a significant 65% reduction in mortality when comparing early treatment with none, along with high levels of heterogeneity. This suggests that early initiation of treatment following symptom onset is key for reducing mortality. We did not detect a significant reduction in mortality associated with preadmission NAI treatment in subsequently hospitalized patients; very few studies were available to address this question, and the absence of data from cases that remained in the community does not allow us to draw conclusions about whether community NAI treatment prevented hospital admission.

### Severe Outcome

Alongside mortality, critical care admission due to influenza is an undesirable outcome of public health importance, worth preventing. Many studies described “severe outcome” using a common definition of critical care admission or mortality, reflecting the occurrence of severe but sometimes survivable A(H1N1)pdm09 infection. It should however be appreciated that some patients with severe disease might have failed to access critical care because of limited availability, which may have introduced bias. Notwithstanding, we observed that NAI treatment (at any time) was associated with a 76% significant increase in the likelihood of severe outcome compared with none. In contrast, a 59% significant reduction in the likelihood of severe outcome was seen for early versus late NAI treatment, but no significant reduction for early NAI treatment versus none. Our data also suggest that preadmission NAIs in patients subsequently hospitalized significantly reduced the likelihood of severe outcome by 49%, albeit based on only 3 studies.

### Pneumonia

Our findings on pneumonia may have been influenced by differential ascertainment and classification of pneumonia. We therefore gave a lower quality score to studies in which information pneumonia ascertainment was not available and performed a subgroup analysis to take this into account (Table 2). We found the likelihood of pneumonia to be significantly increased by 129% for the comparison of any NAI treatment with none, whereas early versus late NAI treatment significantly reduced the likelihood of pneumonia by 65%; we did not find a statistically significant reduction when comparing early treatment versus none.

### Interpretation

Our findings are consistent with earlier data on seasonal influenza, showing that the magnitude of symptomatic benefit due to oseltamivir treatment is increased by early instigation of therapy [8, 26]. We believe the 3 different comparisons in our analyses (treatment at any time vs none, early vs late, and early vs none) help reveal confounding related to treatment propensity but at the same time offer important clinical coherence. We hypothesize that patients with mild illness, more likely to survive and less likely to develop pneumonia, were also less likely to be offered antiviral treatment in most settings during the 2009 pandemic, because of either physician preference or patient care-seeking behavior. Furthermore, we surmise that access to rapid diagnostic testing was variable across settings and that A(H1N1)pdm09 was either not suspected and/or not confirmed in many patients until late in their illness (or late in their admission), by which time either they were recovering or their condition had deteriorated. This may explain the apparent increase in severe outcomes associated with NAI use at any time. It is most likely that those with mild illness who were recovering were left untreated with NAIs and that those with initially mild but later severe illness were treated late as a final attempt at disease reversal. Indeed, unpublished data from the UK FLU-CIN study [27] reveal that among patients with a length of stay ≤4 days (as a proxy for mild to moderate disease) the proportions of patients receiving early, late, or no NAI treatment were 36%, 27%, and 37% respectively, compared with 22%, 41%, and 36% respectively in patients with length of stay >4 days (χ^2^ trend, *P* = .008; data available on request [P. R. M. and J. S. N. V. T., unpublished data].) Thus, comparisons of early treatment versus late may have overestimated treatment effectiveness, whereas comparisons of treatment versus none and early treatment versus none may have underestimated it. In that context, our findings on mortality (early treatment vs none and any treatment vs none), suggest potentially important public health effects because untreated patients were likely to have had milder disease, and our finding of an association between NAI treatment and increased severe outcome seems explainable.

### Limitations

We observed a high degree of heterogeneity among studies examining severe outcome, and although we performed subgroup analyses and stratified by methodological quality, this finding remained largely unexplained. For some of the outcomes we found evidence of publication bias, which may have overestimated the observed pooled effect. All of the studies included in the systematic review were observational designs. This is, in itself, a limitation that cannot be overcome, but it can be argued that such observational data provide a more realistic estimate of the field effectiveness of NAIs in a pandemic situation. Most studies did not provide adjusted risk estimates, but even when these were available there were differences in the extent to which adjustment had been made for potential confounding. Another limitation is the inability to adjust for propensity to treatment. In the absence of random allocation to antivirals, one of the inherent biases in observational studies is the likelihood of receiving treatment. Some of the studies included in the meta-analysis are from low-resource countries and it is likely that treatment was given preferentially to more severely ill patients, thereby underestimating the effectiveness of antiviral therapy in reducing severe outcomes. Finally, a very small proportion of patients received intravenous peramivir (alone or as dual therapy) or dual therapy with oseltamivir and zanamivir. Such patients were well dispersed between studies, and excluding them would have sacrificed too much data. However, because they account for such a small proportion of cases overall, we do not believe they have introduced meaningful bias into the results.

The question of whether NAI treatment has an impact on patient outcomes in a pandemic situation can only ever be answered by using observational data because of the ethical implications of randomization to treatment during a public health emergency. The logical next step is to conduct an individual patient level meta-analysis based on obtaining raw data from observational studies around the world and reanalyzing pooled data [28]. This approach will allow more complete adjustment for confounders, such as comorbid conditions, disease severity, concomitant therapies, propensity for NAI treatment, and the assessment of different NAI treatment regimens.

In conclusion, this systematic review and meta-analysis is to our knowledge the first to examine the effectiveness of NAI treatment solely during the 2009–2010 pandemic, measured against clinical outcomes of likely importance to public health policy-makers. The findings suggest that mortality was reduced among hospitalized patients through early NAI treatment, although the magnitude of benefit offered by early versus late treatment may have been overestimated by treatment propensity. Nevertheless, our finding of a 65% mortality reduction in early treated versus untreated patients suggests a meaningful public health benefit, of relevance to pandemic policy-makers, because it is more likely that untreated cases were less severe rather than more severe and the true effect may therefore have been underestimated. If this is so, pandemic preparedness policies need to emphasize not only the issue of appropriate NAI stockpiling but also practical mechanisms for ensuring easy and early access to treatment during a pandemic.

## Supplementary Data

Supplementary materials are available at *The Journal of Infectious Diseases* online (http://jid.oxfordjournals.org/). Supplementary materials consist of data provided by the author that are published to benefit the reader. The posted materials are not copyedited. The contents of all supplementary data are the sole responsibility of the authors. Questions or messages regarding errors should be addressed to the author.

Supplementary Data
