# Supplementary material for: Impact of Neuraminidase Inhibitor Treatment on Outcomes of Public Health Importance During the 2009–2010 Influenza A(H1N1) Pandemic: A Systematic Review and Meta-Analysis in Hospitalized Patients
Source: J Infect Dis. 2012 Nov 29;207(4):553–63. doi: 10.1093/infdis/jis726 (PMC3549600; doi:10.1093/infdis/jis726)
Supplement: Supplementary Data [file supp_207_4_553__index.html]

Impact of neuraminidase inhibitor treatment on outcomes of public health importance during the 2009-10 influenza A(H1N1) pandemic: a systematic review and meta-analysis in hospitalized patients — Impact of Neuraminidase Inhibitor Treatment on Outcomes of Public Health Importance During the 2009–2010 Influenza A(H1N1) Pandemic: A Systematic Review and Meta-Analysis in Hospitalized Patients — Impact of Neuraminidase Inhibitor Treatment on Outcomes of Public Health Importance During the 2009–2010 Influenza A(H1N1) Pandemic: A Systematic Review and Meta-Analysis in Hospitalized Patients — Supplementary Data 

# Impact of Neuraminidase Inhibitor Treatment on Outcomes of Public Health Importance During the 2009–2010 Influenza A(H1N1) Pandemic: A Systematic Review and Meta-Analysis in Hospitalized Patients

## Supplementary Data

Supplementary Data

**Files in this Data Supplement:**

- Supplementary Table 1 - docx file
- Supplementary Table 2 - docx file
- Supplementary Table 3 - docx file
