# Supplementary material for: Impact of Neuraminidase Inhibitor Treatment on Outcomes of Public Health Importance During the 2009–2010 Influenza A(H1N1) Pandemic: A Systematic Review and Meta-Analysis in Hospitalized Patients
Source: J Infect Dis. 2012 Nov 29;207(4):553–63. doi: 10.1093/infdis/jis726 (PMC3549600; doi:10.1093/infdis/jis726)
Supplement: Supplementary Data [file supp_jis726_jis726supp_table1.docx]

Supplementary Table S1: Search strategy using Ovid Medline

Medline (OVID) – 1996 to present

| \|  \| **Search terms** \| \| --- \| --- \| \| 1. 1. \| exp Influenza A Virus, H1N1 Subtype/ \| \|  \| swine flu.mp. \| \|  \| swine influenza.mp. \| \|  \| (H1N1 pandemic influenza or H1N1v or pandemic influenza 2009).mp. \| \|  \| novel influenza.mp. \| \|  \| H1N1pdm.mp. \| \|  \| (swine-origin influenza or swine-origin type A).tw. \| \|  \| (nH1N1 or pH1N1 or H1N1 or AH1N1).hw. \| \|  \| 1 or 2 or 3 or 4 or 5 or 6 or 7 or 8 \| \|  \| oseltamivir.mp. or exp Oseltamivir/ \| \|  \| zanamivir.mp. or exp Zanamivir/ \| \|  \| exp Neuraminidase/ or neuraminidase inhibitors.mp. \| \|  \| tamiflu.mp. \| \|  \| relenza.mp. \| \|  \| peramivir.mp. \| \|  \| antiviral$.mp. \| \|  \| treatment.mp. \| \|  \| therapy.mp. \| \|  \| 10 or 11 or 12 or 13 or 14 or 15 or 16 or 17 or 18 \| \|  \| 9 and 19 \| \|  \| epidemi$.ti. \| \|  \| pneumonia.mp. \| \|  \| hospital$.mp. \| \|  \| risk factor$.mp. \| \|  \| incidence.mp. \| \|  \| intensive care$.mp. \| \|  \| critical$.mp. \| \|  \| 21 or 22 or 23 or 24 or 25 or 26 or 27 \| \|  \| 9 and 28 \| \|  \| 20 or 29 \| \|  \| remove duplicates from 30 \| \|  \| limit 31 to human \| \|  \| limit 32 to yr="2009 -Current" \| |
| --- | --- | --- | --- | --- | --- | --- | --- | --- | --- | --- | --- | --- | --- | --- | --- | --- | --- | --- | --- | --- | --- | --- | --- | --- | --- | --- | --- | --- | --- | --- | --- | --- | --- | --- | --- | --- | --- | --- | --- | --- | --- | --- | --- | --- | --- | --- | --- | --- | --- | --- | --- | --- | --- | --- | --- | --- | --- | --- | --- | --- | --- | --- | --- | --- | --- | --- | --- | --- |
