# Supplementary material for: Impact of Neuraminidase Inhibitor Treatment on Outcomes of Public Health Importance During the 2009–2010 Influenza A(H1N1) Pandemic: A Systematic Review and Meta-Analysis in Hospitalized Patients
Source: J Infect Dis. 2012 Nov 29;207(4):553–63. doi: 10.1093/infdis/jis726 (PMC3549600; doi:10.1093/infdis/jis726)
Supplement: Supplementary Data [file supp_jis726_jis726supp_table2.docx]

Supplementary Table S2: Summary of 107 studies included in systematic review (SR) and meta-analysis (MA), by outcome measure

| **Author, year (ref)** | **Outcome** | | | | | | **Studies excluded from MA^a^** |
| --- | --- | --- | --- | --- | --- | --- | --- |
|  | **Mortality** | | **Severe outcome** | | **Pneumonia** | |  |
|  | **SR,**  **n=53** | **MA, n=44** | **SR,**  **n=59** | **MA, n=52** | **SR,**  **n=14** | **MA, n=13** | **N =17** |
| Cao et al 2009 [[1](#_ENREF_1)] |  |  |  |  | √ | √ |  |
| Louie et al 2009 [[2](#_ENREF_2)] |  |  | √ | √ | √ | √ |  |
| Dominguez-Cherit et al 2009 [[3](#_ENREF_3)] ^d^ | √ | √ |  |  |  |  |  |
| Echevarria-Zuno et al 2009 [[4](#_ENREF_4)] | √ | √ |  |  |  |  |  |
| Jain et al 2009 [[5](#_ENREF_5)] ^d^ | √ | √ | √ | √ |  |  |  |
| Kwan-Gett et al 2009 [[6](#_ENREF_6)] |  |  | √ | √ |  |  |  |
| Rello et al 2009 [[7](#_ENREF_7)] | √ |  |  |  |  |  | √ |
| Aquino-Esperanza et al 2010 [[8](#_ENREF_8)] | √ | √ | √ | √ |  |  |  |
| Bagdure et al 2010 [[9](#_ENREF_9)]^c^ |  |  | √ | √ |  |  |  |
| Boehringe, 2010 [[10](#_ENREF_10)] |  |  | √ | √ |  |  |  |
| CDC 2010 [[11](#_ENREF_11)] ^c^ |  |  |  |  | √ | √ |  |
| Chien et al 2010 [[12](#_ENREF_12)] |  |  | √ | √ |  |  |  |
| Chitnis et al 2010 [[13](#_ENREF_13)] | √ | √ | √ | √ |  |  |  |
| Creanga et al 2010 [[14](#_ENREF_14)] |  |  | √ |  |  |  | √ |
| Dubar et al 2010 [[15](#_ENREF_15)] |  |  | √ | √ |  |  |  |
| Estenssoro et al 2010 [[16](#_ENREF_16)] | √ | √ |  |  |  |  |  |
| Farias et al 2010 [[17](#_ENREF_17)] | √ | √ |  |  |  |  |  |
| Fuhrman et al 2010 [[18](#_ENREF_18)] |  |  | √ | √ |  |  |  |
| Hernandez-Garcia et al 2010 [[19](#_ENREF_19)] |  |  | √ | √ |  |  |  |
| Jean et al 2010 [[20](#_ENREF_20)] | √ |  |  |  |  |  | √ |
| Koegelenberg et al 2010 [[21](#_ENREF_21)] | √ | √ |  |  |  |  |  |
| Libster et al 2010 [[22](#_ENREF_22)] |  |  | √ | √ |  |  |  |
| Louie et al 2010 [[23](#_ENREF_23)] |  |  | √ |  |  |  | √ |
| Louie et al 2010 [[24](#_ENREF_24)] |  |  | √ | √ |  |  |  |
| Mady et al 2010 [[25](#_ENREF_25)] | √ |  |  |  |  |  | √ |
| Nguyen-Van-Tam et al 2010 [[26](#_ENREF_26)]^b^ | √ | √ | √ | √ |  |  |  |
| O'Riordan et al 2010 [[27](#_ENREF_27)] |  |  | √ | √ |  |  |  |
| Riquelme et al 2010 [[28](#_ENREF_28)] |  |  | √ | √ |  |  |  |
| Rodriguez et al 2010 [[29](#_ENREF_29)] | √ |  |  |  |  |  | √ |
| Santa-Olalla Peralta et al 2010 [[30](#_ENREF_30)] |  |  | √ | √ |  |  |  |
| Siston et al 2010 [[31](#_ENREF_31)]^d^ | √ | √ | √ | √ |  |  |  |
| Souza et al 2010 [[32](#_ENREF_32)] | √ | √ | √ | √ |  |  |  |
| Stein et al 2010 [[33](#_ENREF_33)] |  |  | √ | √ | √ | √ |  |
| To et al 2010 [[34](#_ENREF_34)] |  |  |  |  |  |  | √ |
| Vasoo et al 2010 [[35](#_ENREF_35)] |  |  | √ | √ |  |  |  |
| Wada et al 2010 [[36](#_ENREF_36)] | √ | √ |  |  |  |  |  |
| Xi et al 2010 [[37](#_ENREF_37)] | √ | √ |  |  |  |  |  |
| Yang et al 2010 [[38](#_ENREF_38)] | √ | √ | √ | √ |  |  |  |
| Yates et al 2010 [[39](#_ENREF_39)] |  |  | √ | √ |  |  |  |
| Akinci et al 2011 [[40](#_ENREF_40)] |  |  | √ | √ | √ |  |  |
| Altmann et al 2011 [[41](#_ENREF_41)] | √ | √ |  |  |  |  |  |
| Choi et al 2011 [[42](#_ENREF_42)] ^c d^ | √ | √ |  |  |  |  |  |
| Choi W.I et al 2011 [[43](#_ENREF_43)] ^c d^ | √ | √ |  |  |  |  |  |
| Chudasama et al 2011 [[44](#_ENREF_44)] |  |  | √ | √ |  |  |  |
| Creanga et al 2011 [[45](#_ENREF_45)] |  |  | √ | √ |  |  |  |
| Ellington et al 2011 [[46](#_ENREF_46)] | √ |  |  |  |  |  | √ |
| Fuhrman et al 2011 [[47](#_ENREF_47)] |  |  | √ | √ |  |  |  |
| Hasegawa et al 2011 [[48](#_ENREF_48)] |  |  |  |  |  |  | √ |
| Hiba et al 2011 [[49](#_ENREF_49)] | √ | √ | √ | √ | √ | √ |  |
| Ismail et al 2011 [[50](#_ENREF_50)] | √ | √ |  |  |  |  |  |
| Jeon et al 2011 [[51](#_ENREF_51)] |  |  |  |  | √ | √ |  |
| Kendirli et al 2011 [[52](#_ENREF_52)] | √ | √ |  |  |  |  |  |
| Launes et al 2011 [[53](#_ENREF_53)] |  |  | √ | √ |  |  |  |
| Louie et al 2011 [[54](#_ENREF_54)] | √ |  |  |  |  |  | √ |
| Lucker et al 2011 [[55](#_ENREF_55)] |  |  | √ | √ |  |  |  |
| Maravi-Poma et al 2011 [[56](#_ENREF_56)] |  |  |  |  |  |  | √ |
| Meschi et al 2011 [[57](#_ENREF_57)] |  |  |  |  | √ | √ |  |
| Mickiene et al 2011 [[58](#_ENREF_58)] | √ | √ | √ | √ |  |  |  |
| Moral et al 2011 [[59](#_ENREF_59)] |  |  | √ | √ |  |  |  |
| Nakai et al 2011 [[60](#_ENREF_60)] |  |  |  |  | √ | √ |  |
| Oh et al 2011 [[61](#_ENREF_61)] ^c d^ |  |  | √ | √ |  |  |  |
| Poeppl et al 2011 [[62](#_ENREF_62)] | √ | √ | √ | √ |  |  |  |
| Schellongowski et al 2011 [[63](#_ENREF_63)] | √ | √ |  |  |  |  |  |
| Skarbinski et al 2011 [[64](#_ENREF_64)] ^c d^ |  |  | √ | √ |  |  |  |
| Thompson et al 2011 [[65](#_ENREF_65)] | √ | √ | √ | √ |  |  |  |
| Viasus et al 2011 [[66](#_ENREF_66)] |  |  | √ | √ |  |  |  |
| Viasus et al 2011 [[67](#_ENREF_67)] |  |  | √ | √ |  |  |  |
| Viasus et al 2011 [[68](#_ENREF_68)] | √ |  | √ |  |  |  | √ |
| Yokota et al 2011 [[69](#_ENREF_69)] | √ | √ |  |  |  |  |  |
| Yu et al 2011 [[70](#_ENREF_70)] |  |  | √ | √ |  |  |  |
| Zheng et al 2011 [[71](#_ENREF_71)] |  |  | √ | √ |  |  |  |
| Campbell et al 2011 [[72](#_ENREF_72)] | √ | √ | √ | √ |  |  |  |
| Custodio et al 2011 [[73](#_ENREF_73)] ^d^ |  |  | √ | √ |  |  |  |
| Del Rosal et al 2011 [[74](#_ENREF_74)] |  |  | √ | √ |  |  |  |
| Gonzalez-Velez et al 2011 [[75](#_ENREF_75)] |  |  |  |  | √ | √ | √ |
| Higuera Iglesias et al 2011 [[76](#_ENREF_76)] |  |  |  |  | √ | √ |  |
| Hsann et al 2011 [[77](#_ENREF_77)] |  |  | √ | √ |  |  |  |
| Newsome et al 2011 [[78](#_ENREF_78)] | √ | √ |  |  |  |  |  |
| Rhim et al 2011 [[79](#_ENREF_79)] |  |  |  |  | √ | √ |  |
| Louie et al 2011 [[80](#_ENREF_80)] |  |  | √ |  |  |  | √ |
| Viasus et al 2011 [[81](#_ENREF_81)] |  |  |  |  | √ | √ |  |
| Rodriguez et al 2011 [[82](#_ENREF_82)] | √ | √ |  |  |  |  |  |
| Riquelme et al 2011 [[83](#_ENREF_83)] | √ | √ |  |  |  |  |  |
| Lee et al 2010 [[84](#_ENREF_84)] | √ | √ |  |  |  |  |  |
| Louie et al 2009 [[85](#_ENREF_85)] | √ | √ |  |  |  |  |  |
| Da Dalt et al 2011 [[86](#_ENREF_86)] |  |  | √ | √ |  |  |  |
| Randolph et al 2011 [[87](#_ENREF_87)] |  |  | √ |  |  |  | √ |
| Perez-Padilla et al 2009 [[88](#_ENREF_88)] | √ | √ |  |  |  |  |  |
| Chudasama et al 2010 [[89](#_ENREF_89)] |  |  | √ |  |  |  | √ |
| Chudasama et al 2010 [[90](#_ENREF_90)] | √ | √ |  |  |  |  |  |
| Chudasama et al 2010 [[91](#_ENREF_91)] |  |  | √ |  |  |  | √ |
| Santa-Olalla Peralta et al 2010 [[92](#_ENREF_92)] | √ | √ |  |  |  |  |  |
| Gilca et al 2011 [[93](#_ENREF_93)] | √ | √ | √ | √ |  |  |  |
| Javadi et al 2011 [[94](#_ENREF_94)] | √ | √ |  |  |  |  |  |
| Kedia et al 2011 [[95](#_ENREF_95)] ^c d^ |  |  | √ | √ |  |  |  |
| Martin-Loeches et al 2011 [[96](#_ENREF_96)] | √ | √ |  |  |  |  |  |
| Miranda-Choque et al 2011 [[97](#_ENREF_97)] | √ | √ |  |  |  |  |  |
| Qiao et al 2011 [[98](#_ENREF_98)] | √ | √ |  |  |  |  |  |
| Yung et al 2011 [[99](#_ENREF_99)] | √ | √ |  |  |  |  |  |
| Cortes Garcia et al 2012 [[100](#_ENREF_100)] |  |  | √ | √ |  |  |  |
| Figueiro-Filho et al 2012 [[101](#_ENREF_101)] | √ | √ | √ | √ | √ | √ |  |
| Mady et al 2012 [[102](#_ENREF_102)] | √ | √ |  |  |  |  |  |
| Rhim et al 2012 [[103](#_ENREF_103)] |  |  |  |  | √ | √ |  |
| Yang et al 2012 [[104](#_ENREF_104)] | √ | √ | √ | √ |  |  |  |
| Low et al 2010 [[105](#_ENREF_105)]^b^ |  |  | √ | √ |  |  |  |
| Peters et al 2011 [[106](#_ENREF_106)] |  |  | √ | √ |  |  |  |
| Tabarsi et al 2011 [[107](#_ENREF_107)]^b^ | √ | √ |  |  |  |  |  |

SR, systematic review; MA , meta-analysis

^a^ Reasons for exclusions are outlined in supplementary Table 3

^b^ authors provided additional data on NAI use

^c^ provided information on combined oseltamivir and peramivir use

^d^ reported combined use of NAI and non-NAI (rimantadine, amantadine or ribavarin) therapy

**References used:**

1. Cao B, Li X-W, Mao Y, et al. Clinical features of the initial cases of 2009 pandemic influenza A (H1N1) virus infection in China. N Engl J Med **2009**; 361:2507-17.

2. Louie J, Winter K, Harriman K, et al. Hospitalized Patients With Novel Influenza A (H1N1) Virus Infection-California, April-May, 2009 (Reprinted from MMWR, vol 58, pg 536-541, 2009). Jama-Journal of the American Medical Association **2009**; 302:137-40.

3. Dominguez-Cherit G, Lapinsky SE, Macias AE, et al. Critically Ill patients with 2009 influenza A(H1N1) in Mexico. Jama **2009**; 302:1880-7.

4. Echevarria-Zuno S, Mejia-Arangure JM, Mar-Obeso AJ, et al. Infection and death from influenza A H1N1 virus in Mexico: a retrospective analysis. Lancet **2009**; 374:2072-9.

5. Jain S, Kamimoto L, Bramley AM, et al. Hospitalized patients with 2009 H1N1 influenza in the United States, April-June 2009. N Engl J Med **2009**; 361:1935-44.

6. Kwan-Gett TS, Baer A, Duchin JS. Spring 2009 H1N1 influenza outbreak in King County, Washington. Disaster med **2009**; 3 Suppl 2:S109-16.

7. Rello J, Rodriguez A, Ibanez P, et al. Intensive care adult patients with severe respiratory failure caused by Influenza A (H1N1)v in Spain. Crit Care **2009**; 13:R148.

8. Aquino-Esperanza J, Rodriguez PO, Boughen S, et al. Severe respiratory disease in an intensive care unit during influenza A(H1N1)2009 pandemia. Medicina **2010**; 70 (5):401-7.

9. Bagdure D, Curtis DJ, Dobyns E, Glode MP, Dominguez SR. Hospitalized children with 2009 pandemic influenza A (H1N1): Comparison to seasonal influenza and risk factors for admission to the ICU. PLoS ONE **2010**; 5 (12).

10. Boehringer C. Novel influenza A 2009-A comparison of intensive care unit vs non-intensive care unit patients. International Journal of Infectious Diseases **2010**; 14:E93-E.

11. Centers for Disease C, Prevention. 2009 pandemic influenza A (H1N1) in pregnant women requiring intensive care - New York City, 2009. MMWR Morb Mortal Wkly Rep **2010**; 59:321-6.

12. Chien Y-S, Su C-P, Tsai H-T, et al. Predictors and outcomes of respiratory failure among hospitalized pneumonia patients with 2009 H1N1 influenza in Taiwan. J Infect **2010**; 60:168-74.

13. Chitnis AS, Truelove SA, Druckenmiller JK, Heffernan RT, Davis JP. Epidemiologic and clinical features among patients hospitalized in Wisconsin with 2009 H1N1 influenza A virus infections, April to August 2009. Wmj **2010**; 109:201-8.

14. Creanga AA, Johnson TF, Graitcer SB, et al. Severity of 2009 pandemic influenza A (H1N1) virus infection in pregnant women. Obstet Gynecol **2010**; 115:717-26.

15. Dubar G, Azria E, Tesniere A, et al. French experience of 2009 A/H1N1v influenza in pregnant women. PLoS ONE [Electronic Resource] **2010**; 5.

16. Estenssoro E, Rios FG, Apezteguia C, et al. Pandemic 2009 influenza A in Argentina: a study of 337 patients on mechanical ventilation. Am J Respir Crit Care Med **2010**; 182:41-8.

17. Farias JA, Fernandez A, Monteverde E, et al. Critically ill infants and children with influenza A (H1N1) in pediatric intensive care units in Argentina. Intensive Care Medicine **2010**; 36:1015-22.

18. Fuhrman C, Bonmarin I, Paty AC, et al. Severe hospitalised 2009 pandemic influenza A(H1N1) cases in France, 1 July-15 November 2009. Euro Surveill **2010**; 15:14.

19. Hernandez-Garcia I, Garcia-Iglesias MA, Lopez-Garcia E, Saenz-Gonzalez MDC. [Epidemiological characteristics of hospitalized patients with influenza caused by A(H1N1) 2009 virus]. Gac Sanit **2010**; 24:501-2.

20. Jean C, Louie JK, Glaser CA, et al. Invasive group A streptococcal infection concurrent with 2009 H1N1 influenza. Clin Infect Dis **2010**; 50:e59-62.

21. Koegelenberg CFN, Irusen EM, Cooper R, et al. High mortality from respiratory failure secondary to swine-origin influenza A (H1N1) in South Africa. Qjm **2010**; 103:319-25.

22. Libster R, Bugna J, Coviello S, et al. Pediatric hospitalizations associated with 2009 pandemic influenza A (H1N1) in Argentina. N Engl J Med **2010**; 362:45-55.

23. Louie JK, Acosta M, Jamieson DJ, Honein MA, California Pandemic Working G. Severe 2009 H1N1 influenza in pregnant and postpartum women in California. N Engl J Med **2010**; 362:27-35.

24. Louie JK, Gavali S, Acosta M, et al. Children hospitalized with 2009 novel influenza A(H1N1) in California. Arch Pediatr Adolesc Med **2010**; 164:1023-31.

25. Mady A, Ramadan OS, Yousef A, Amr AA, Kherallah M. CLINICAL EXPERIENCE WITH SEVERE 2009 H1N1 INFLUENZA IN INTENSIVE CARE UNIT AT KING SAUD MEDICAL COMPLEX, SAUDI ARABIA. Intensive Care Medicine **2010**; 36:0206.

26. Nguyen-Van-Tam JS, Openshaw PJM, Hashim A, et al. Risk factors for hospitalisation and poor outcome with pandemic A/H1N1 influenza: United Kingdom first wave (May-September 2009). Thorax **2010**; 65:645-51.

27. O'Riordan S, Barton M, Yau Y, Read SE, Allen U, Tran D. Risk factors and outcomes among children admitted to hospital with pandemic H1N1 influenza. Cmaj **2010**; 182:39-44.

28. Riquelme R, Riquelme M, Rioseco ML, et al. Characteristics of hospitalised patients with 2009 H1N1 influenza in Chile. Eur Respir J **2010**; 36:864-9.

29. Rodriguez A, Zaragoza R, Diaz E, et al. EARLY OSELTAMIVIR TREATMENT WAS ASSOCIATED WITH IMPROVED OUTCOMES IN 2009 PANDEMIC INFLUENZA A (H1N1)V IN SPAIN. Intensive Care Medicine **2010**; 36:S136.

30. Santa-Olalla Peralta P, Cortes-Garcia M, Vicente-Herrero M, et al. Risk factors for disease severity among hospitalised patients with 2009 pandemic influenza A (H1N1) in Spain, April - December 2009. Euro Surveill **2010**; 15:23.

31. Siston AM, Rasmussen SA, Honein MA, et al. Pandemic 2009 influenza A(H1N1) virus illness among pregnant women in the United States. Jama **2010**; 303:1517-25.

32. Souza TML, Salluh JIF, Bozza FA, et al. H1N1pdm influenza infection in hospitalized cancer patients: clinical evolution and viral analysis. PLoS ONE [Electronic Resource] **2010**; 5:e14158.

33. Stein M, Tasher D, Glikman D, et al. Hospitalization of children with influenza A(H1N1) virus in Israel during the 2009 outbreak in Israel: a multicenter survey. Arch Pediatr Adolesc Med **2010**; 164:1015-22.

34. To KKW, Hung IFN, Li IWS, et al. Delayed clearance of viral load and marked cytokine activation in severe cases of pandemic H1N1 2009 influenza virus infection. Clin Infect Dis **2010**; 50:850-9.

35. Vasoo S, Crank CW, Singh K. Timely administration of antivirals for pandemic (H1N1) 2009 influenza. Clin Infect Dis **2010**; 50:1428-9.

36. Wada K, Nishiura H, Kawana A. An epidemiological analysis of severe cases of the influenza A (H1N1) 2009 virus infection in Japan. Influenza other respi **2010**; 4:179-86.

37. Xi X, Xu Y, Jiang L, et al. Hospitalized adult patients with 2009 influenza A(H1N1) in Beijing, China: risk factors for hospital mortality. BMC Infectious Diseases **2010**; 10:256.

38. Yang P, Deng Y, Pang X, et al. Severe, critical and fatal cases of 2009 H1N1 influenza in China. J Infect **2010**; 61:277-83.

39. Yates L, Pierce M, Stephens S, et al. Influenza A/H1N1v in pregnancy: an investigation of the characteristics and management of affected women and the relationship to pregnancy outcomes for mother and infant. Health Technol Assess **2010**; 14:109-82.

40. Akinci E, Kayaaslan B, Yetkin MA, et al. Analysis of 113 hospitalized patients with confirmed 2009 influenza a (H1N1) virus infection. [Turkish]. Turkish Journal of Medical Sciences **2011**; 41 (3):507-14.

41. Altmann M, Fiebig L, Soyka J, von Kries R, Dehnert M, Haas W. Severe cases of pandemic (H1N1) 2009 in children, Germany. Emerging Infectious Diseases **2011**; 17:186-92.

42. Choi EY, Huh JW, Lim CM, et al. Critically ill patients with pandemic influenza A/H1N1 2009 at a Medical Center in Korea. Tuberculosis and Respiratory Diseases **2011**; 70 (1):28-35.

43. Choi WI, Yim JJ, Park J, et al. Clinical characteristics and outcomes of H1N1-associated pneumonia among adults in South Korea. Int J Tuberc Lung Dis **2011**; 15:270-5.

44. Chudasama RK, Patel UV, Verma PB, et al. Clinico-epidemiological features of the hospitalized patients with 2009 pandemic influenza A (H1N1) virus infection in Saurashtra region, India (September, 2009 to February, 2010). Lung India **2011**; 28 (1):11-5.

45. Creanga AA, Kamimoto L, Newsome K, et al. Seasonal and 2009 pandemic influenza A (H1N1) virus infection during pregnancy: A population-based study of hospitalized cases. American Journal of Obstetrics and Gynecology **2011**; 204 (6 SUPPL.):S38-S45.

46. Ellington SR, Hartman LK, Acosta M, et al. Pandemic 2009 influenza A (H1N1) in 71 critically ill pregnant women in California. American Journal of Obstetrics and Gynecology **2011**; 204 (6 SUPPL.):S21-S30.

47. Fuhrman C, Bonmarin I, Bitar D, et al. Adult intensive-care patients with 2009 pandemic influenza A(H1N1) infection. Epidemiol Infect **2011**; 139:1202-9.

48. Hasegawa M, Okada T, Sakata H, et al. Pandemic (H1N1) 2009-associated pneumonia in children, Japan. Emerging Infectious Diseases **2011**; 17:279-82.

49. Hiba V, Chowers M, Levi-Vinograd I, Rubinovitch B, Leibovici L, Paul M. Benefit of early treatment with oseltamivir in hospitalized patients with documented 2009 influenza A (H1N1): Retrospective cohort study. Journal of Antimicrobial Chemotherapy **2011**; 66 1150-5.

50. Ismail HIM, Tan KK, Lee YL, et al. Characteristics Of children hospitalized for pandemic (H1N1) 2009, Malaysia. Emerging Infectious Diseases **2011**; 17 (4):708-10.

51. Jeon MH, Chung JW, Choi SH, Kim TH, Lee EJ, Choo EJ. Pneumonia risk factors and clinical features of hospitalized patients older than 15 years with pandemic influenza A (H1N1) in South Korea: A multicenter study. Diagnostic Microbiology and Infectious Disease **2011**; 70 (2):230-5.

52. Kendirli T, Demirkol D, Yildizdacs D, et al. Critically ill children with pandemic influenza (H1N1) in pediatric intensive care units in Turkey. Pediatr Crit Care Med **2011**.

53. Launes C, Garcia-Garcia JJ, Jordan I, Martinez-Planas A, Selva L, Munoz-Almagro C. 2009 Influenza A H1N1 Infections: Delays in Starting Treatment With Oseltamivir Were Associated With a More Severe Disease. Pediatr Infect Dis J **2011**; 30:622-5.

54. Louie JK, Acosta M, Samuel MC, et al. A novel risk factor for a novel virus: obesity and 2009 pandemic influenza A (H1N1). Clin Infect Dis **2011**; 52:301-12.

55. Lucker LM, Kherad O, Iten A, et al. Clinical features and outcomes of hospitalised adults and children with the 2009 influenza A H1N1 infection at Geneva's University Hospital. Swiss Medical Weekly **2011**; 141.

56. Maravi-Poma E, Martin-Loeches I, Regidor E, et al. Severe 2009 A/H1N1v influenza in pregnant women in Spain. Critical Care Medicine **2011**; 39 (5):945-51.

57. Meschi S, Selleri M, Lalle E, et al. Duration of viral shedding in hospitalized patients infected with pandemic H1N1. BMC Infectious Diseases **2011**; 11:1-10.

58. Mickiene A, Daniuseviciute L, Vanagaite N, et al. Hospitalized Adult Patients with 2009 Pandemic Influenza A (H1N1) in Kaunas, Lithuania. Medicina-Lithuania **2011**; 47:11-8.

59. Moral L, Marco N, Toral T, Fuentes MJ, Fuentes L, Lillo L. Burden of Severe 2009 Pandemic Influenza A (H1N1) Infection in Children in Southeast Spain. Enferm Infecc Microbiol Clin **2011**; 29:497-501.

60. Nakai A, Minakami H, Unno N, et al. Characteristics of pregnant Japanese women who required hospitalization for treatment of pandemic (H1N1) 2009--low mortality rate may be due to early antiviral use. J Infect **2011**; 62:232-3.

61. Oh WS, Lee SJ, Lee CS, et al. A Prediction Rule to Identify Severe Cases among Adult Patients Hospitalized with Pandemic Influenza A (H1N1) 2009. Journal of Korean Medical Science **2011**; 26:499-506.

62. Poeppl W, Hell M, Herkner H, et al. Clinical aspects of 2009 pandemic influenza A (H1N1) virus infection in Austria. Infection **2011**; 39:341-52.

63. Schellongowski P, Ullrich R, Hieber C, et al. A surge of flu-associated adult respiratory distress syndrome in an Austrian tertiary care hospital during the 2009/2010 Influenza A H1N1v pandemic. Wiener Klinische Wochenschrift **2011**; 123 (7-8):209-14.

64. Skarbinski J, Jain S, Bramley A, et al. Hospitalized patients with 2009 pandemic influenza A (H1N1) virus infection in the United States--September-October 2009. Clin Infect Dis **2011**; 52 Suppl 1:S50-9.

65. Thompson DL, Jungk J, Hancock E, et al. Risk Factors for 2009 Pandemic Influenza A (H1N1)-Related Hospitalization and Death Among Racial/Ethnic Groups in New Mexico. American journal of public health **2011**; 101:1776-84.

66. Viasus D, Pano-Pardo JR, Cordero E, et al. Effect of immunomodulatory therapies in patients with pandemic influenza A (H1N1) 2009 complicated by pneumonia. J Infect **2011**; 62:193-9.

67. Viasus D, Pano-Pardo JR, Pachon J, et al. Factors associated with severe disease in hospitalized adults with pandemic (H1N1) 2009 in Spain. Clinical Microbiology and Infection **2011**; 17 (5):738-46.

68. Viasus D, Pano-Pardo JR, Pachon J, et al. Timing of Oseltamivir Administration and Outcomes in Hospitalized Adults with Pandemic 2009 Influenza A (H1N1) Virus Infection. Chest **2011**.

69. Yokota RTC, Skalinski LM, Igansi CN, et al. Risk factors for death from pandemic (H1N1) 2009, southern Brazil. Emerging Infectious Diseases **2011**; 17:1467-71.

70. Yu H, Feng Z, Uyeki TM, et al. Risk factors for severe illness with 2009 pandemic influenza A (H1N1) virus infection in China. Clin Infect Dis **2011**; 52:457-65.

71. Zheng Y, He Y, Deng J, et al. Hospitalized children with 2009 influenza A (H1N1) infection in Shenzhen, China, november-december 2009. Pediatric Pulmonology **2011**; 46 (3):246-52.

72. Campbell CNJ, Mytton OT, McLean EM, et al. Hospitalization in two waves of pandemic influenza A(H1N1) in England. Epidemiol Infect **2011**; 139:1560-9.

73. Custodio HT, Gayle MO, Bailey CS, Wludyka PS, Rathore MH. Comparison of ICU and non-ICU patients infected with the 2009 H1N1 influenza virus in a Florida Children's hospital between April and December 2009. Eastern Journal of Medicine **2011**; 16:188-93.

74. Del Rosal T, Baquero-Artigao F, Calvo C, et al. Pandemic H1N1 influenza-associated hospitalizations in children in Madrid, Spain. Influenza and other Respiratory Viruses **2011**; 5:e544-e51.

75. Gonzalez-Velez AE, Diaz-Agero-Perez C, Robustillo-Rodela A, et al. Factors associated to admission to Intensive Care in patients hospitalized due to pandemic Influenza A/H1N1 2009. [Spanish]. Medicina Intensiva **2011**; 35:463-9.

76. Higuera Iglesias AL, Kudo K, Manabe T, et al. Reducing occurrence and severity of pneumonia due to pandemic H1N1 2009 by early oseltamivir administration: a retrospective study in Mexico. PLoS ONE [Electronic Resource] **2011**; 6.

77. Hsann YM, Thang SP, Abdul Salam ZH, Yang Y, Sui-Leong Lim V, Yang KS. Clinical characteristics and outcomes of hospitalized patients with 2009 H1N1 influenza in a large acute care tertiary hospital, Singapore. American Journal of Infection Control **2011**; 39:e49-e51.

78. Newsome K, Williams J, Way S, et al. Maternal and infant outcomes among severely ill pregnant and postpartum women with 2009 pandemic influenza A (H1N1) - United States, April 2009-August 2010. Morbidity and Mortality Weekly Report **2011**; 60:1193-6.

79. Rhim JW, Lee KY, Youn YS, Kang JH, Kim JC. Epidemiological and clinical characteristics of childhood pandemic 2009 H1N1 virus infection: An observational cohort study. BMC Infectious Diseases **2011**; 11.

80. Louie JK, Jamieson DJ, Rasmussen SA. 2009 pandemic influenza A (H1N1) virus infection in postpartum women in California. American Journal of Obstetrics and Gynecology **2011**; 204 (2):144.e1-.e6.

81. Viasus D, Pano-Pardo JR, Pachon J, et al. Pneumonia complicating pandemic (H1N1) 2009: Risk factors, clinical features, and outcomes. Medicine **2011**; 90:328-36.

82. Rodriguez A, Diaz E, Martin-Loeches I, et al. Impact of early oseltamivir treatment on outcome in critically ill patients with 2009 pandemic influenza A. Journal of Antimicrobial Chemotherapy **2011**; 66 1140-49.

83. Riquelme R, Jimenez P, Videla AJ, et al. Predicting mortality in hospitalized patients with 2009 H1N1 influenza pneumonia. International Journal of Tuberculosis and Lung Disease **2011**; 15 (4):542-6.

84. Lee EH, Wu C, Lee EU, et al. Fatalities associated with the 2009 H1N1 influenza A virus in New York city. Clin Infect Dis **2010**; 50:1498-504.

85. Louie JK, Acosta M, Winter K, et al. Factors associated with death or hospitalization due to pandemic 2009 influenza A(H1N1) infection in California. Jama **2009**; 302:1896-902.

86. Da Dalt L, Chillemi C, Cavicchiolo ME, et al. Pandemic influenza A (H1N1v) infection in pediatric population: a multicenter study in a north-east area of Italy. Ital **2011**; 37:24.

87. Randolph AG, Vaughn F, Sullivan R, et al. Critically ill children during the 2009-2010 influenza pandemic in the United States. Pediatrics **2011**; 128:e1450-8.

88. Perez-Padilla R, de la Rosa-Zamboni D, Ponce de Leon S, et al. Pneumonia and respiratory failure from swine-origin influenza A (H1N1) in Mexico. N Engl J Med **2009**; 361:680-9.

89. Chudasama RK, Patel UV, Verma PB. Hospitalizations associated with 2009 influenza A (H1N1) and seasonal influenza in Saurashtra region, India. J **2010**; 4:834-41.

90. Chudasama RK, Patel UV, Verma PB, et al. Characteristics of fatal cases of pandemic influenza A (H1N1) from September 2009 to January 2010 in Saurashtra Region, India. Online Journal of Health and Allied Sciences **2010**; 9.

91. Chudasama RK, Verma PB, Amin CD, Gohel B, Savariya D, Ninama R. Correlates of severe disease in patients admitted with 2009 pandemic influenza A (H1N1) infection in Saurashtra region, India. Indian Journal of Critical Care Medicine **2010**; 14:113-20.

92. Santa-Olalla Peralta P, Cortes Garcia M, Limia Sanchez A, et al. [Critically ill patients with 2009 pandemic influenza A (H1N1) infection in Spain: factors associated with death, April 2009-January 2010]. Rev Esp Salud Publica **2010**; 84:547-67.

93. Gilca R, de Serres G, Boulianne N, et al. Risk factors for hospitalization and severe outcomes of 2009 pandemic H1N1 influenza in Quebec, Canada. Influenza and other Respiratory Viruses **2011**; 5:247-55.

94. Javadi AA, Ataei B, Khorvash F, et al. Clinical features of novel 2009 influenza a (H1N1) infection in Isfahan, Iran. Journal of Research in Medical Sciences **2011**; 16:1550-4.

95. Kedia S, Stroud B, Parsons J, et al. Pediatric neurological complications of 2009 pandemic influenza A (H1N1). Arch Neurol **2011**; 68:455-62.

96. Martin-Loeches I, Diaz E, Vidaur L, et al. Pandemic and post-pandemic Influenza A (H1N1) infection in critically ill patients. Critical Care **2011**; 15.

97. Miranda-Choque E, Ramirez C, Candela-Herrera J, et al. Children hospitalized with influenza pneumonia AH1N1/2009 pandemic in the INSN

Ninos hospitalizados con neumonia por influenza AH1N1/2009 pandemico en un hospital de referencia de peru. Revista Peruana de Medicina de Experimental y Salud Publica **2011**; 28:610-6.

98. Qiao C, Zhao J, Mao M. Effect of early antiviral treatment on perinatal prognosis of the pregnant women with severe H1N1 influenza virus infection. Maternal and Child Health Care of China **2011**; 26:1566-9.

99. Yung M, Slater A, Festa M, et al. Pandemic H1N1 in children requiring intensive care in Australia and New Zealand during winter 2009. Pediatrics **2011**; 127:e156-63.

100. Cortes Garcia M, Sierra Moros MJ, Santa-Olalla Peralta P, Hernandez-Barrera V, Jimenez-Garcia R, Pachon I. Clinical characteristics and outcomes of diabetic patients who were hospitalised with 2009 pandemic influenza A H1N1 infection. J Infect **2012**; 64:218-24.

101. Figueiro-Filho EA, Oliveira MLG, Pompilio MA, et al. Obstetric, clinical, and perinatal implications of H1N1 viral infection during pregnancy. International Journal of Gynecology and Obstetrics **2012**; 116:214-8.

102. Mady A, Ramadan OS, Yousef A, Mandourah Y, Amr AA, Kherallah M. Clinical experience with severe 2009 H1N1 influenza in the intensive care unit at King Saud Medical City, Saudi Arabia. Journal of Infection and Public Health **2012**; 5:52-6.

103. Rhim JW, Go EJ, Lee KY, et al. Pandemic 2009 H1N1 virus infection in children and adults: A cohort study at a single hospital throughout the epidemic. International archives of medicine **2012**; 5:13.

104. Yang Sg, Cao B, Liang Lr, et al. Antiviral therapy and outcomes of patients with pneumonia caused by influenza a pandemic (H1N1) virus. PLoS ONE [Electronic Resource] **2012**; 7.

105. Low CY, Kee T, Chan KP, et al. Pandemic (H1N1) 2009 infection in adult solid organ transplant recipients in Singapore. Transplantation **2010**; 90:1016-21.

106. Peters PJ, Skarbinski J, Louie JK, et al. HIV-infected hospitalized patients with 2009 pandemic influenza A (pH1N1)--United States, spring and summer 2009. Clin Infect Dis **2011**; 52 Suppl 1:S183-8.

107. Tabarsi P, Moradi A, Marjani M, et al. Factors associated with death or intensive care unit admission due to pandemic 2009 influenza A (H1N1) infection. Annals of Thoracic Medicine **2011**; 6 (2):91-5.
