# Supplementary material for: Impact of Neuraminidase Inhibitor Treatment on Outcomes of Public Health Importance During the 2009–2010 Influenza A(H1N1) Pandemic: A Systematic Review and Meta-Analysis in Hospitalized Patients
Source: J Infect Dis. 2012 Nov 29;207(4):553–63. doi: 10.1093/infdis/jis726 (PMC3549600; doi:10.1093/infdis/jis726)
Supplement: Supplementary Data [file supp_jis726_jis726supp_table3.docx]

Supplementary Table S3: Reasons for rejection of 17 articles unsuitable for meta-analysis

| 1. **Unable to determine sample size of control population not treated with antivirals**   Randolph et al 2011 [1] | |
| --- | --- |
| 1. **Assessed other exposure definitions** | |
| ***Author (year)*** | ***Exposure assessed :*** |
| Viasus et al 2011 [[2](#_ENREF_2)] | Oseltamivir administration (+ 1-d increase). This study also overlaps with references [66, 67 in Table S2] used in the meta-analysis |
| 1. **Examined other influenza outcomes/complications** | |
| ***Author (year)*** | ***Outcome assessed:*** |
| Hasegawa et al 2011[[3](#_ENREF_3)] | Complications following pandemic (H1N1) 2009–associated pneumonia |
| To et al 2010 [[4](#_ENREF_4)] | ARDS and/ died vs survived without ARDS |
| Maravi-Poma et al 2011 [[5](#_ENREF_5)] | Compares pregnant and non-pregnant women affected by influenza A/H1N1v and admitted to ICU. This study also overlaps with references. This study also overlaps with reference [82 in Table S2] used in meta-analysis |
| 1. **Duplicate/ overlapping population** | |
| ***Rejected article: Author (year)*** | ***Alternate article used in meta-analysis: Author (year)*** |
| Rello et al 2009 [[6](#_ENREF_6)] | Rodriguez et al 2011 [82 in Table S2] |
| Chudasama et al 2010 [[7](#_ENREF_7)] | Chudasama et al 2011 [44 in Table S2] |
| Chudasama et al 2010 [[8](#_ENREF_8)] | Chudasama et al 2011 [44 in Table S2] |
| Creanga et al 2010 [[9](#_ENREF_9)] | Siston et al 2010 [31 in Table S2] |
| Jean et al 2010 [[10](#_ENREF_10)] | Louie et al 2009 [85 in Table S2] |
| Louie et al 2010 [[11](#_ENREF_11)] | Siston et al 2010 [31 in Table S2] |
| Mady et al 2010 [[12](#_ENREF_12)] | Mady et al 2012 [102 in Table S2] |
| Rodriguez et al 2010 [[13](#_ENREF_13)] | Rodriguez et al 2011 [82 in Table S2] |
| Ellington et al 2011 [[14](#_ENREF_14)] | Newsome et al 2011 [78 in Table S2] |
| Gonzalez-Velez et al 2011 [[15](#_ENREF_15)] | Santa-Olalla Peralta et al 2010 [30 in Table S2] |
| Louie et al 2011 [[16](#_ENREF_16)] | Louie et al 2009 [85 in Table S2] |
| Louie et al 2011[[17](#_ENREF_17)] | Siston et al 2010 [31 in Table S2] |

**Rejected references**

1. Randolph, A.G., et al., *Critically ill children during the 2009-2010 influenza pandemic in the United States.* Pediatrics, 2011. **128**(6): p. e1450-8.

2. Viasus, D., et al., *Timing of Oseltamivir Administration and Outcomes in Hospitalized Adults with Pandemic 2009 Influenza A (H1N1) Virus Infection.* Chest, 2011.

3. Hasegawa, M., et al., *Pandemic (H1N1) 2009-associated pneumonia in children, Japan.* Emerging Infectious Diseases, 2011. **17**(2): p. 279-82.

4. To, K.K.W., et al., *Delayed clearance of viral load and marked cytokine activation in severe cases of pandemic H1N1 2009 influenza virus infection.* Clinical Infectious Diseases, 2010. **50**(6): p. 850-9.

5. Maravi-Poma, E., et al., *Severe 2009 A/H1N1v influenza in pregnant women in Spain.* Critical Care Medicine, 2011. **39 (5)**: p. 945-951.

6. Rello, J., et al., *Intensive care adult patients with severe respiratory failure caused by Influenza A (H1N1)v in Spain.* Critical Care (London, England), 2009. **13**(5): p. R148.

7. Chudasama, R.K., et al., *Hospitalizations associated with 2009 influenza A (H1N1) and seasonal influenza in Saurashtra region, India.* Journal of Infection in Developing Countries, 2010. **4**(12): p. 834-41.

8. Chudasama, R.K., et al., *Correlates of severe disease in patients admitted with 2009 pandemic influenza A (H1N1) infection in Saurashtra region, India.* Indian Journal of Critical Care Medicine, 2010. **14**(3): p. 113-120.

9. Creanga, A.A., et al., *Severity of 2009 pandemic influenza A (H1N1) virus infection in pregnant women.* Obstetrics & Gynecology, 2010. **115**(4): p. 717-26.

10. Jean, C., et al., *Invasive group A streptococcal infection concurrent with 2009 H1N1 influenza.* Clinical Infectious Diseases, 2010. **50**(10): p. e59-62.

11. Louie, J.K., et al., *Severe 2009 H1N1 influenza in pregnant and postpartum women in California.* New England Journal of Medicine, 2010. **362**(1): p. 27-35.

12. Mady, A., et al., *CLINICAL EXPERIENCE WITH SEVERE 2009 H1N1 INFLUENZA IN INTENSIVE CARE UNIT AT KING SAUD MEDICAL COMPLEX, SAUDI ARABIA.* Intensive Care Medicine, 2010. **36**: p. 0206.

13. Rodriguez, A., et al., *EARLY OSELTAMIVIR TREATMENT WAS ASSOCIATED WITH IMPROVED OUTCOMES IN 2009 PANDEMIC INFLUENZA A (H1N1)V IN SPAIN.* Intensive Care Medicine, 2010. **36**: p. S136.

14. Ellington, S.R., et al., *Pandemic 2009 influenza A (H1N1) in 71 critically ill pregnant women in California.* American Journal of Obstetrics and Gynecology, 2011. **204 (6 SUPPL.)**: p. S21-S30.

15. Gonzalez-Velez, A.E., et al., *Factors associated to admission to Intensive Care in patients hospitalized due to pandemic Influenza A/H1N1 2009. [Spanish].* Medicina Intensiva, 2011. **35**(8): p. 463-469.

16. Louie, J.K., et al., *A novel risk factor for a novel virus: obesity and 2009 pandemic influenza A (H1N1).* Clinical Infectious Diseases, 2011. **52**(3): p. 301-12.

17. Louie, J.K., et al., *2009 pandemic influenza A (H1N1) virus infection in postpartum women in California.* American Journal of Obstetrics and Gynecology, 2011. **204 (2)**: p. 144.e1-144.e6.
